# Coagulation abnormalities in Dengue fever infection: A systematic review and meta-analysis

**DOI:** 10.1371/journal.pntd.0009666

**Published:** 2021-08-18

**Authors:** Tiruneh Adane, Solomon Getawa

**Affiliations:** Department of Hematology and Immunohematology, School of Biomedical and Laboratory Sciences, College of Medicine and Health Sciences, University of Gondar, Gondar, Ethiopia; University of Glasgow, UNITED KINGDOM

## Abstract

**Background:**

Coagulation mechanisms are reported to be affected in dengue illness and evidenced by prolonged activated partial thromboplastin time (APTT) and prothrombin time (PT). The main aim of this systematic review and meta-analysis is to determine the magnitude of coagulation abnormalities among patients with dengue fever infection.

**Method:**

This systematic review and meta-analysis were conducted per the Preferred Reporting Items for Systematic Reviews and Meta-Analyses (PRISMA) guideline. The Joana Brigg’s Institute (JBI) critical appraisal checklist was used for quality appraisal. STATA version 11 software was used for meta-analysis. The magnitude of coagulation abnormalities among dengue fever patients was determined by using a random-effects model. Subgroup and sensitivity analysis were performed to investigate the possible source of heterogeneity. Egger weighted regression tests were used to check the presence of publication bias among the included articles.

**Result:**

Forty-two studies with a total of 12,221 dengue fever patients were eligible for meta-analysis in this study. Of which 22, 15, and 26 studies were used to determine the magnitude of prolonged APTT, PT, and thrombocytopenia, respectively. The magnitude of prolonged APTT and PT among patients with dengue fever infection were 42.91% (95% CI: 30.95, 54.87) I^2^ = 99.1% and 16.48% (95% CI: 10.95, 22.01) I^2^ = 97.0%, respectively. Besides, the magnitude of thrombocytopenia among dengue fever patients was 70.29% (95% CI: 62.69, 77.89) I^2^ = 99.3%. The magnitude of prolonged APTT in children and adults was 51.21% (95% CI: 24.54, 77.89) and 44.89% (95% CI: 28.32, 61.45), respectively. Similarly, the overall magnitude of prolonged PT in children and adults were 13.40% (95% CI: 6.09, 20.71) and 18.73% (95% CI: 7.49, 29.96), respectively.

**Conclusion:**

The result of this study showed that there is a high magnitude of prolonged APTT and PT in dengue fever patients. Therefore, screening and early correction of coagulation abnormalities may be helpful to reduce further complications in those patients.

## Author summary

Coagulation mechanisms are reported to be affected in dengue illness and evidenced by prolonged activated partial thromboplastin time (APTT) and prothrombin time (PT). The magnitude of prolonged APTT and PT among patients with dengue fever infection were 42.91% (95% CI: 30.95, 54.87) I^2^ = 99.1% and 16.48% (95% CI: 10.95, 22.01) I^2^ = 97.0%, respectively. The magnitude of thrombocytopenia among dengue fever patients was 70.29% (95% CI: 62.69, 77.89) I^2^ = 99.3%.Screening and early correction of coagulation abnormalities may be helpful to reduce further complications in those patients.

## Introduction

Dengue is transmitted by the bite of an infected Aedes mosquito. The female Aedes mosquito gets infected with the dengue virus after sucking blood from an infected person during acute febrile illness [1]. Dengue illness is currently the most important mosquito-borne viral disease in the tropical areas of the world [2]. It is caused by one of the four dengue virus serotypes (DEN-1, DEN-2, DEN-3, and DEN-4) and Aedes aegypti is the main vector [3]. The fifth and latest addition to the existing serotypes of dengue viruses is DENV-5 which has been announced in October 2013 [4]. According to estimates of the World Health Organization (WHO), about 50 million cases of Dengue fever (DF) occur annually worldwide and 2.5 billion people live in risk areas [5]. Every year about 50–100 million cases of dengue infection, 500,000 cases of Dengue hemorrhagic fever (DHF) and at least 12,000 deaths occur worldwide; ninety percent of these deaths occur in children less than 15 years of age [6,7].

The DF is classically a self-limiting, nonspecific illness characterized by fever, headache, myalgia, and constitutional symptoms. DHF is a more serious clinical entity. The WHO classifies DHF in four grades (I to IV). The DHF grades I and II represent relatively mild cases without shock, whereas grade III and IV cases are more severe and accompanied by shock [8]. Although DF is a self-limited febrile illness, DHF is characterized by prominent hemorrhagic manifestations with thrombocytopenia, increased vascular permeability, and is associated with a high mortality rate [9]. The primary pathophysiologic abnormality seen in DHF is an acute increase in vascular permeability that leads to plasma leakage into the extravascular compartment [10]. The WHO defines dengue shock syndrome (DSS) as DHF plus signs of circulatory failure manifested by rapid and weak pulse, narrow pulse pressure (≤20 mmHg) or hypotension for age, prolonged capillary refill, cold and clammy skin, and restlessness [1]. Initial infection with a particular serotype (the primary infection) is usually asymptomatic or results in mild disease manifestations. However, subsequent infection (secondary dengue infections) may lead to severe disease which manifests in the form of DHF/DSS [11].

The clinical picture of DF shows abnormal hemostatic activities, which is demonstrated by thrombocytopenia [12]. Thrombocytopenia may occur in DF/DHF as a result of either decreased production (bone marrow suppression) and/or increased peripheral destruction [3]. Platelet destruction may occur as a result of complement activation and also because of peripheral sequestration. Hemorrhage may be a consequence of thrombocytopenia and associated platelet dysfunction or disseminated intravascular coagulation (DIC) [13]. Thrombocytopenia is common in DHF and is one of the criteria stipulated by the WHO for the clinical case definition, but it has also been noted in up to 50% of cases of DF [14]. The appearance of IgM antiplatelet antibodies destroying platelets is a predictor for the development of thrombocytopenia. An increased level of platelet-associated IgM during the acute phase of secondary infection was associated with the development of DHF [15].

Initial hemostasis is tightly linked to inflammation. Inflammation-induced during infection generally shifts the hemostatic mechanism toward thrombosis by upregulation of procoagulant factors, down-regulation of anticoagulants, and inhibit fibrinolytic activity [16]. The activated partial thromboplastin time (APTT) and prothrombin time (PT) are screening assays used for the initial assessment of disorders of hemostasis [17]. Coagulation and anticoagulation mechanisms are reported to be affected and evidenced by prolonged APTT, PT, hyperfibrinogenemia, and decreased fibrin monomers [18]. Dysfunction of the damaged liver might be responsible for the decreased synthesis of specific factors in the intrinsic pathway. Increased factor consumption is also associated with APTT prolongation, but in a less significant manner [10]. Dengue viral infection induces the endothelial production of tissue plasminogen activator as well as IL-6. IL-6 can down-regulate the synthesis of coagulation factor XII the first factor to initiate the intrinsic pathway of the coagulation cascade [19].

The main aim of this systematic review and meta-analysis is to determine the magnitude of coagulation abnormalities among dengue fever infection globally. This may help to give insight for the concerned bodies to design appropriate intervention plans and also the early treatment of the patients since dengue fever is one of the neglected diseases.

## Methods

### Design

This systematic review and meta-analysis were conducted following the PRISMA guideline [20] (S1 PRISMA Checklist).

### Eligibility criteria

All studies that reported the magnitude of coagulation abnormalities and also thrombocytopenia among dengue fever patients using the English language and published in the peer-reviewed journal were included. Case-controls, cross-sectional, retrospective cohort, and prospective studies were also included in this study. The study also included articles that reported the magnitude of thrombocytopenia among dengue fever patients without the reports of PT and/or APTT. Studies that reported the coagulation profile of dengue patients in the form of a continuous variable (mean ± standard deviation) were also included in this study. There is no age restriction in the population types included in this study. Review articles, abstracts, editorials, commentaries, and poster presentations were excluded from this study.

### Search strategy

PubMed, Cochrane Library, Scopus, Google Scholar, and African Journals Online were the major databases used to review all published articles. The search for published studies was not restricted by time, and all published articles up to March 2021 were included in this review. Reference lists of retrieved articles were searched to identify any studies that are not retrieved from electronic databases. The search terms were used separately and in combination using Boolean operators like “OR” or “AND”. The search terms used were coagulation abnormalities, coagulation profiles, hematological profiles, partial thromboplastin time, prothrombin time, prolonged APTT, prolonged PT, hemostatic derangement, thrombocytopenia, dengue fever, dengue hemorrhagic fever, and dengue shock syndrome (S1 PubMed Search strategy).

### Study selection and quality appraisal

All retrieved articles were imported to EndNote X7 (Thomson Reuters, USA). After excluding duplications, titles and/or abstracts of articles were independently screened by two authors (TA and SG). The authors agreed to settle their argument through discussion. Then, articles that comply with the eligibility criteria and are sufficiently valid for our research question underwent full-text appraisal. JBI critical appraisal checklist for simple prevalence, cohort, and case-control studies was used for quality appraisal using 9, 11, and 10 criteria, respectively. For each question, a score was assigned (0 for ‘not reported or not appropriate’ and 1 for ‘yes); the scores were summarized across the items to attain a total score that ranged from 0 to 9, 0 to 10, and 0 to 11 for simple prevalence, case-control, and cohort studies, respectively. Studies were then classified as having a low, medium, and high quality based on the awarded points. Articles having high and medium quality were included in the final analysis (S1 Quality Appraisal).

### Data extraction

Relevant studies that fulfilled the eligibility criteria were subjected to data extraction and summarized into an excel spreadsheet. Information extracted from the included studies were; the name of the first author, year of study, country, publication year, study design, sample size, the magnitude of thrombocytopenia, prolonged PT, and prolonged APTT (Table 1).

### Meta-analysis

STATA version 11 software was used for meta-analysis. Random-effects model was used to determine the magnitude of coagulation abnormalities along with 95% confidence intervals (CIs). The I^2^ statistics were used to assess the magnitude of heterogeneity from the included articles. The I^2^ value of 25, 50, and 75 indicates low, medium, and high heterogeneity, respectively [21]. Subgroup and sensitivity analysis were performed to explore the possible source of heterogeneity. Eggers test and funnel plot were used to check the presence of publication bias among the included articles. A P-value <0.05 in Egger’s test was considered to be evidence of statistically significant publication bias [22].

## Result

### Selection of studies

**Fig 1:**
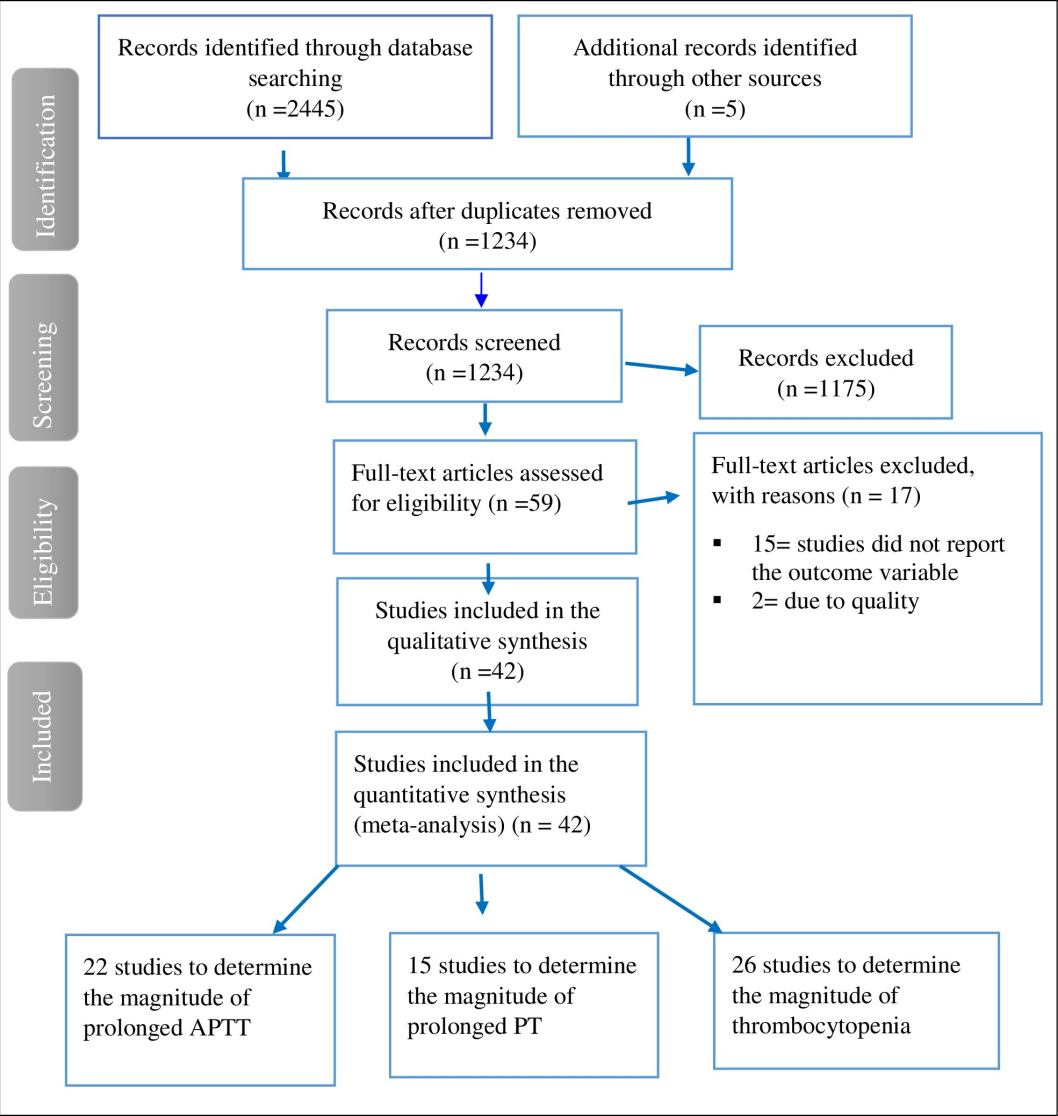
Flow chart to describe the selection of studies for the systematic review and meta-analysis on the magnitude of coagulation abnormalities among dengue fever patients.

Of the 2450 articles assessed initially for full-text analysis, 42 studies were included in the final meta-analysis. Of the total, 1216 articles were excluded due to duplication and 1175 unrelated articles were excluded by their title and abstract. The remaining 59 full-text articles were assessed for inclusion; of them, 17 full-text articles were excluded with reason. (*Fig 1*).

### Characteristics of included studies

Forty-two studies were included in this study. In total, 23 studies were in India, 8 in Pakistan, 3 in Taiwan, 2 in Brazil, and 2 in Malaysia. In Saudi Arabia, Indonesia, Sri Lanka, and Sudan, 4 articles one from each country have reported coagulation abnormalities in dengue virus infected patients. The number of patients with dengue infection ranges from 24 to 2022, both of them were in India. The total number of study participants in this systematic review and meta-analysis was 12,221. With regard to the types of populations, 12 studies were conducted in adults, 13 studies were in children, 14 studies were in all age groups, and the other 3 studies did not explicitly specify their target populations. Ten studies reported the magnitude of both prolonged APTT, prolonged PT, and thrombocytopenia while 20 studies reported the magnitude of both prolonged APTT and PT among dengue fever patients. Twenty-six studies reported the magnitude of thrombocytopenia without the reports of PT and/or APTT among dengue fever patients (Table 1).

**Table 1:** Characteristics of included studies.

Author, year of publication	Year of study	Country	Sample size	Study design	Populations	Prolonged PT	Prolonged APTT	Thrombocytopenia
Pranesh 2020 [13]	2018–2019	India	100	Case-control	Adults	49	43	69
Barbosa et al 2019 [17]	2003–2007	Brazil	187	Retrospective	All age group	34.2	65.8	-
Hassan et al 2018 [18]	2013–2016	Pakistan	200	Observational	Adults	13.6	21.6	-
Vijayaraghavan et al 2020 [19]	2005–2010	Malaysia	203	Retrospective	Children	-	51.85	56.4
Kannan et al 2014 [23]	NR	India	264	NR	NR	-	22.3	-
Balakrishnan et al 2017 [24]	2013–2014	India	306	Descriptive	Children	20.9	91.1	-
Yashaswini et al 2017 [25]	2016	India	100	Observational	Adults	6	38	-
Kadadavar et al 2019 [26]	2016–2018	India	100	Prospective	Adults	-	36	97
Dhooria et al 2008 [27]	2005–2006	India	81	Retrospective	Children	3.7	-	100
Kavitha et al 2020 [28]	2016	India	128	Cross-sectional	All age group	-	86.1	89
Kalori et al 2011 [29]	2010	India	356	Cross-sectional	All age group	-	24	89
Hamsa et al 2019 [30]	2017	India	170	Prospective	Adults	-	64.7	25.9
Jameel et al 2012 [31]	2010	Pakistan	364	Cross-sectional	NR	24	25	-
Ali et al 2007 [32]	2001–2006	Pakistan	210	Retrospective	All age group	2.5	16.7	77.1
Khalil et al 2014 [33]	2008–2010	Pakistan	532	Retrospective	Adults	12	42.3	98.12
Mallhi et al 2017 [34]	2008–2013	Malaysia	667	Retrospective	Adults	33.3	23.8	59.2
Ayyub et al 2006 [35]	2004–2005	Saudi Arabia	80	Prospective	All age group	0	25.64	58.97
Budastra et al 2009 [36]	2007	Indonesia	131	Prospective	Children	15.26	16.03	-
Liu et al 2013 [37]	2002	Taiwan	100	NR	Adults	1.5	89.7	100
Kulasinghe et al 2016 [38]	2013	Sri Lanka	384	Prospective	Children	-	67	-
Bashir et al 2015 [39]	2013–2014	Sudan	334	Prospective	All age group	9	12.6	83.5
Khan et al 2020 [40]	2018–2019	Pakistan	310	Cross-sectional	NR	6.3	25.26	48
Shah et al 2005 [41]	2004	India	69	Prospective	Children	24.1	55.9	50
Ghalige et al 2014 [42]	2010–2012	India	100	Observational	Children	15.5 ±1.3	36.9 ±2.4	43
Selvan et al 2015 [43]	2015	India	300	Prospective	Children	-	-	92
Khan et al 2014 [44]	2011–2012	Pakistan	250	Retrospective	All age group	-	-	65.2
Tulara et al 2019 [45]	2017	India	112	Prospective	Adults	-	-	97
Kumari et al 2020 [46]	NR	India	210	Observational	Children	-	-	99.52
Tong et al 2007 [47]	2003	India	24	Prospective	All age group	-	-	87.5
Castilho et al 2020 [48]	2014	Brazil	387	Retrospective	All age group	-	-	40.3
Rai et al 2019 [49]	2016–2018	India	2022	Prospective	All age group	-	-	62.6
Tewari et al 2018 [50]	2013	India	443	Observational	All age group	-	-	67
Chairulfatah et al 2003 [51]	1995–1996	India	1300	Retrospective	All age group	-	-	58
Khan et al 2014 [52]	2011–2012	Pakistan	107	Retrospective	All age group	-	-	71
Patel et al 2020 [53]	NR	India	80	Descriptive	Adults	-	-	85
Almas et al 2010 [54]	2007	Pakistan	699	Cross-sectional	Adults	13.02±4.62	36.50±12.28	-
Prabhavathi et al 2017 [55]	NR	India	100	Prospective	Children	12.4 ± 1.1	26.7 ± 4.1	-
Hsieh et al 2016 [56]	2015	Taiwan	75	Retrospective	Adults	-	44.9±11.1	-
Kumar et al 2017 [57]	2015	India	306	Descriptive	Children	19±3.7	46±7	-
Ho et al 2013 [58]	2007	Taiwan	100	Retrospective	Children	-	40 ± 45	-
Bandaru et al 2019 [59]	2019	India	105	Prospective	Children	16.8 ± 7.9	48.3 ± 21.2	-
Vinoj M 2019 [60]	2019	India	100	Prospective	All age group	-	45.22 ±7.08	-

NR: not reported/appropriate

### The magnitude of prolonged APTT and PT in dengue fever infection

**Fig 2:**
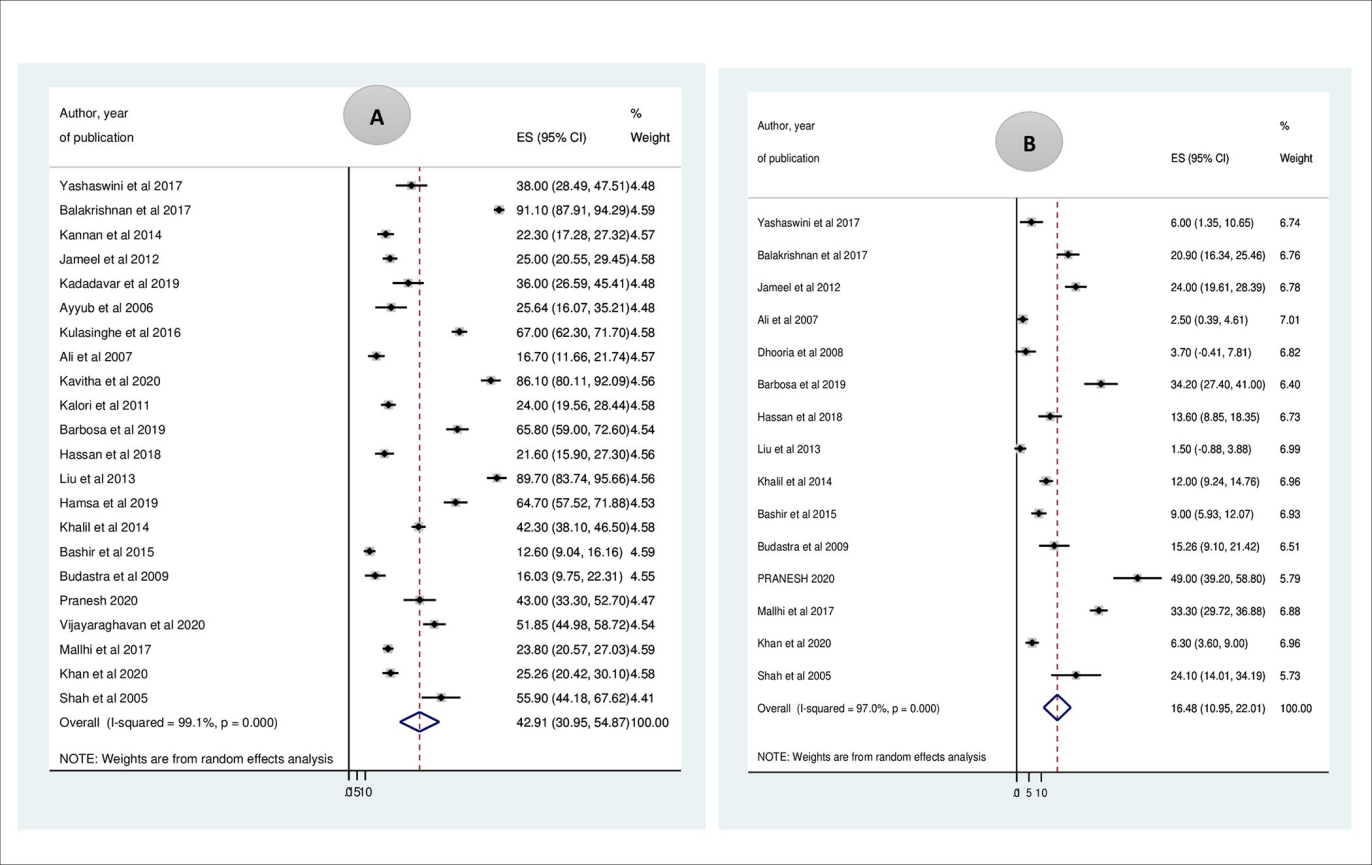
Forest plot displaying A. The magnitude of prolonged APTT among dengue fever patients B. The magnitude of prolonged PT among dengue fever patients.

Using 22 studies conducted in different countries of the world, the magnitude of prolonged APTT in patients with dengue fever was 42.91% (95% CI: 30.95, 54.87) I^2^ = 99.1%. The minimum and maximum magnitude of prolonged APTT were 12.6% [39] and 91.10% [24] in Indonesia and India, respectively. In this review, 15 studies were included to determine the magnitude of prolonged PT among dengue fever patients. The minimum and maximum time prolongation of PT were 1.5% [37] and 49% [13] in Taiwan and India, respectively. Accordingly, the overall magnitude of prolonged PT was 16.48% (95% CI: 10.95, 22.01) I^2^ = 97.0% (*Fig 2*).

Nine studies [41,42,54–60] were used to summarize prolonged APTT in the form of mean± SD in patients with dengue fever. Accordingly, the summarized mean of APTT in the included studies was 41.65 ±7.39 (95% CI: 41.29, 42.01). Besides, using 6 studies [41,42,54,55,57,59], the mean PT value was 15.17±2.47 (95% CI: 15.04, 15.30).

### Magnitude of thrombocytopenia in dengue fever patients

**Fig 3:**
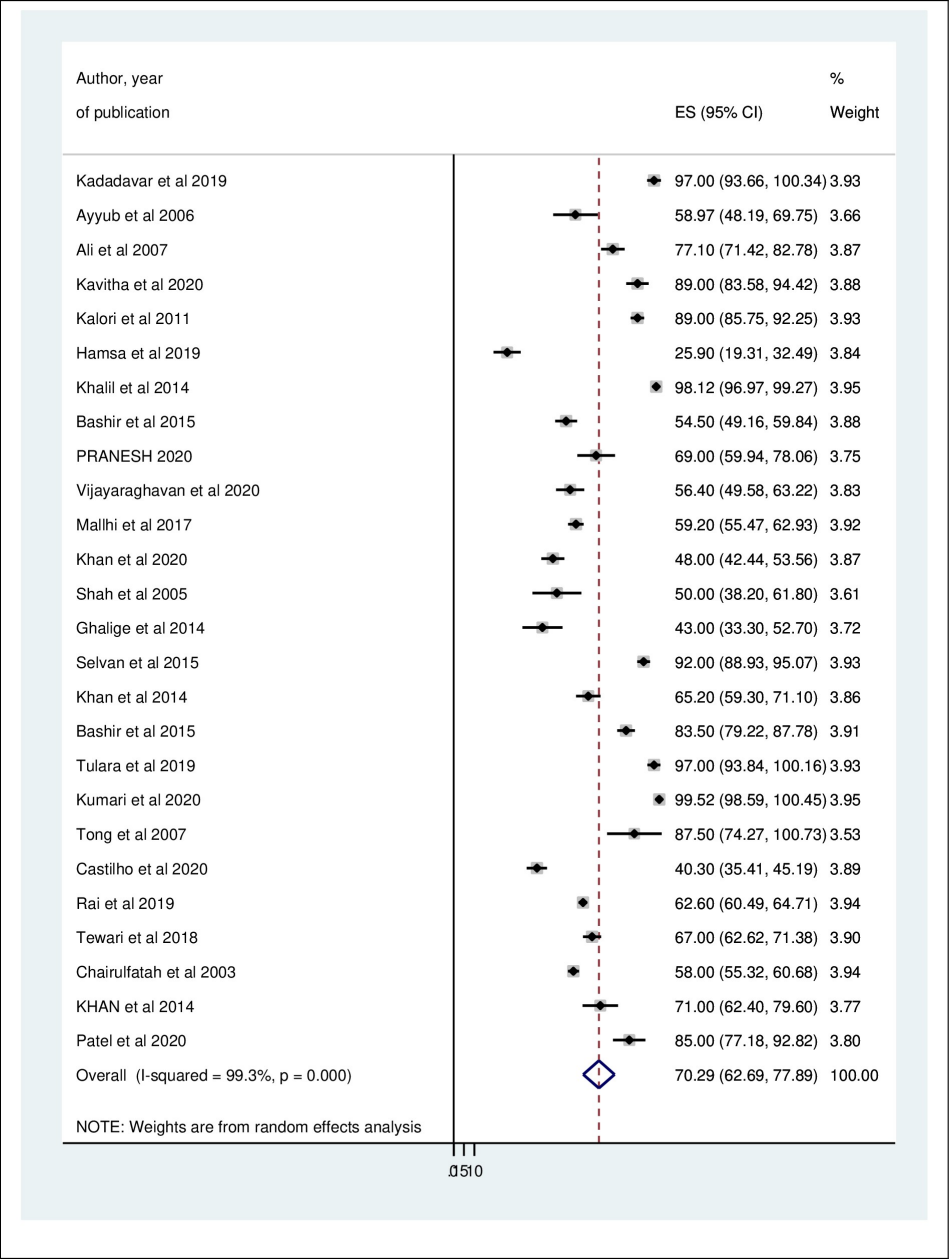
Forest plot displaying the magnitude of thrombocytopenia among dengue fever patients.

A total of 26 studies were evaluated to determine the overall magnitude of thrombocytopenia in dengue fever patients. The lowest and highest magnitude of thrombocytopenia among the included studies was 25.9% in Saudi Arabia [30] and 97.00% [33] in India, respectively. The overall magnitude of thrombocytopenia among dengue fever patients was 70.29% (95% CI: 62.69, 77.89) I^2^ = 99.3% (*Fig 3*).

### Sub-group analysis based on target populations

**Fig 4:**
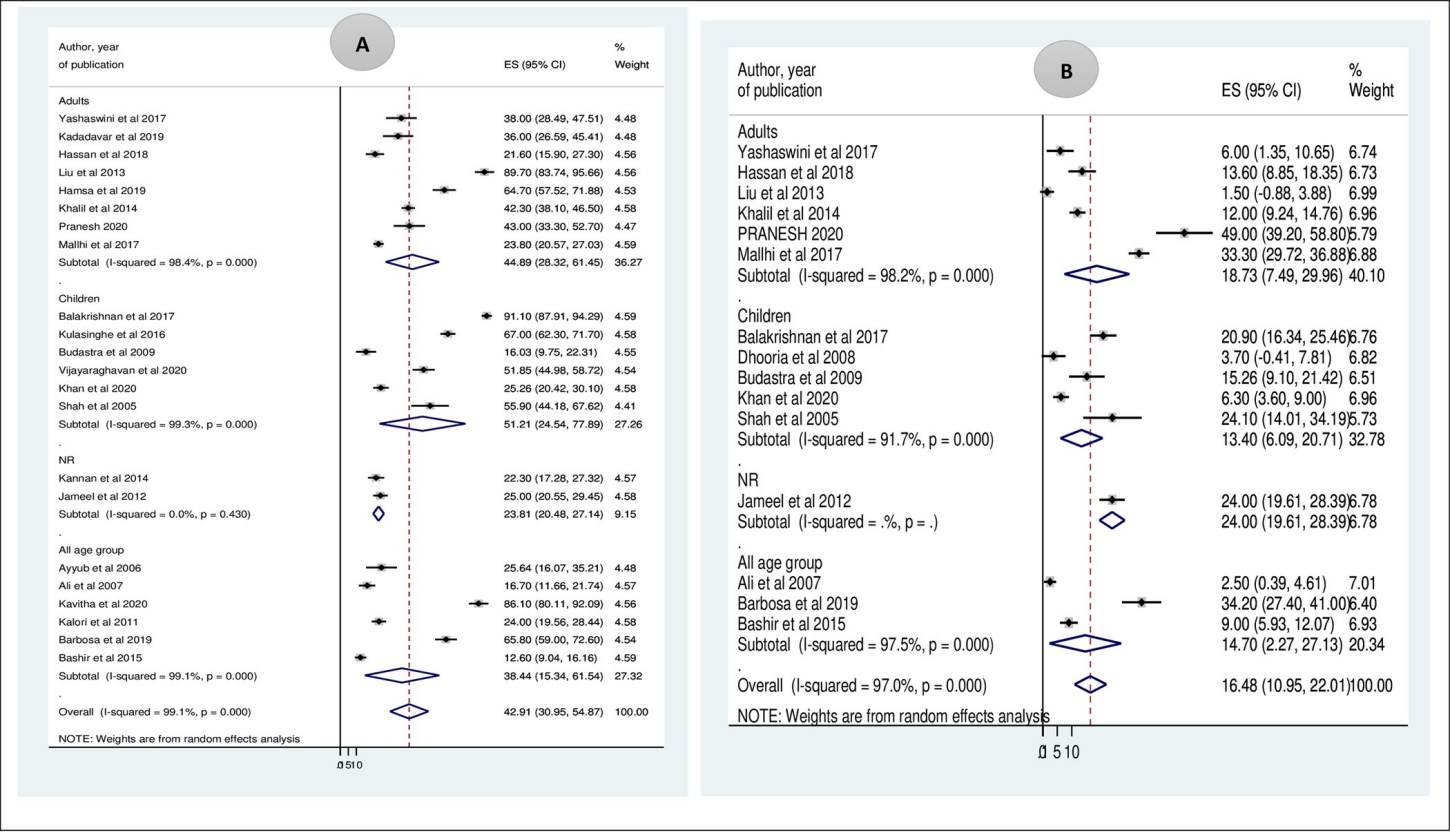
A Sub-group analysis of prolonged APTT based on age distribution B. Sub-group analysis of prolonged PT based on age distribution.

To investigate the possible source of heterogeneity, we have done a sub-group analysis using the target populations of the included studies (children, adults, all age groups, and age group not specified). The magnitude of prolonged APTT in children, adults, all age groups, and age group not specified (NR) was 51.21% (95% CI: 24.54, 77.89), 44.89% (95% CI: 28.32, 61.45), 38.44% (95% CI: 11.34, 61.54), and 23.81 (95% CI: 20.48, 27.14) respectively. Similarly, the pooled time prolongation of PT in children, adults, and all age groups was 13.40% (95% CI: 6.09, 20.71), 18.73% (95% CI: 7.49, 29.96), and 14.70% (95% CI: 2.27, 27.13), respectively. The I^2^ test indicated high heterogeneity both in prolonged APTT (I^2^ = 99.1%, (P<0.001) and prolonged PT (I^2^ = 97.0%, P<0.001) (*Fig 4*).

### Publication bias

**Fig 5:**
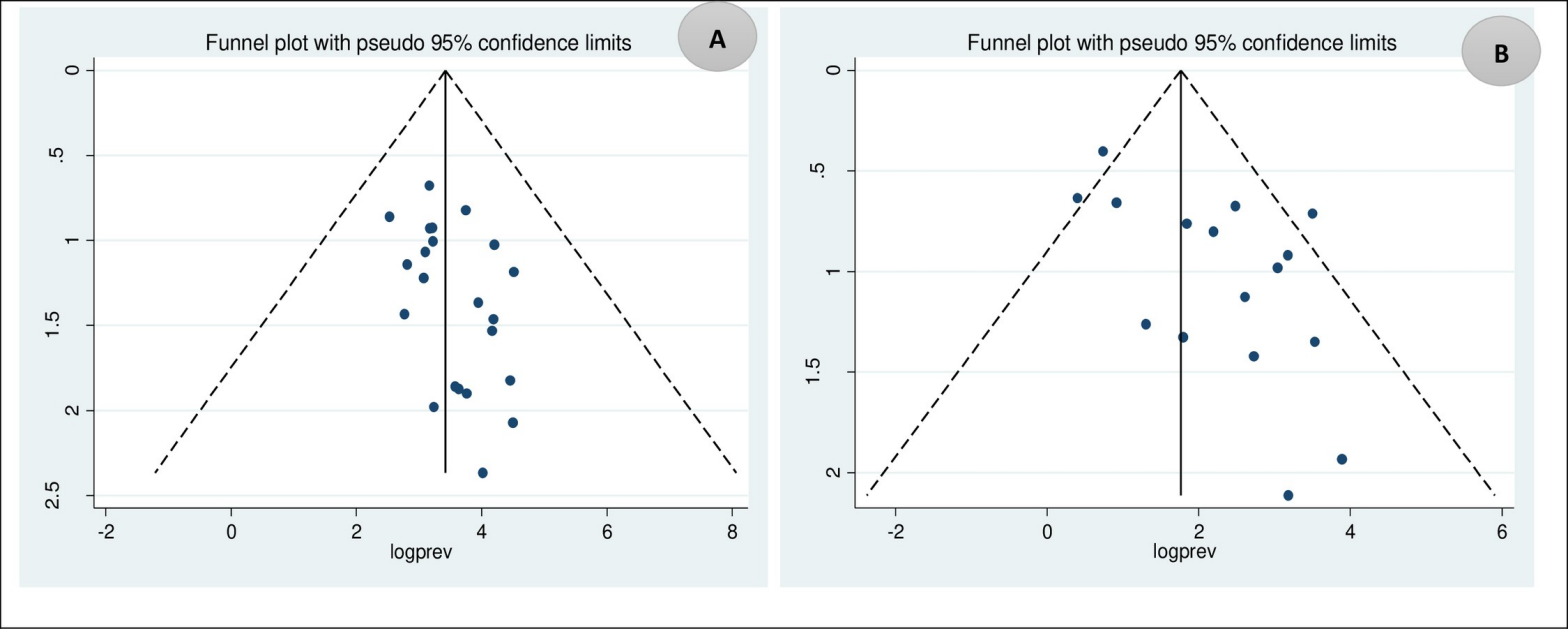
Funnel plot of included studies A. on the magnitude of APTT dengue fever patients B. on the magnitude of PT dengue fever patients.

The presence of publication bias was determined statistically by the Eggers test and visually by funnel plot. The result showed that there is no significant publication bias among studies included to determine the magnitude of prolonged APTT (p-value = 0.883). However, there is significant publication bias among the included studies to determine the magnitude of prolonged PT (p-value = 0.001) (*Fig 5*).

### Trim and fill analysis

Since we have detected significant publication bias, trim and fill analysis was done to overcome the impact of the small-study effect. Six additional studies were filled to the model, and the overall magnitude of prolonged PT in the random-effect model were found to be 6.09% (95% CI: -0.64, 12.82).

### Sensitivity analysis

Since there is a high level of heterogeneity in the included studies, a sensitivity analysis was done to assess the effect of each study on the overall result. However, the result of the analysis revealed that the individual studies don’t affect the overall magnitude of coagulation abnormalities as indicated in Tables 2 and 3.

**Table 2:** Sensitivity analysis to estimate the effect each study on the magnitude of prolonged APTT among patients with dengue virus infection.

Study omitted	Point Estimate	95% Conf. Interval		Heterogeneity	
		Lower	Upper	I^2^	P-Value
Pranesh 2020 [13]	42.90	30.58	55.23	99.2%	≤0.001
Barbosa et al 2019 [17]	41.82	29.52	54.12	99.2%	≤0.001
Hassan et al 2018 [18]	43.93	31.56	56.30	99.2%	≤0.001
Vijayaraghavan et al 2020 [19]	42.48	30.08	54.88	99.2%	≤0.001
Balakrishnan et al 2017 [24]	40.56	30.71	50.41	98.5%	≤0.001
Kadadavar et al 2019 [26]	43.23	30.90	55.26	99.2%	≤0.001
Kavitha et al 2020 [28]	40.84	28.94	52.75	99.1%	≤0.001
Kalori et al 2011 [29]	43.82	31.32	56.31	99.1%	≤0.001
Hamsa et al 2019 [30]	41.87	29.57	54.18	99.2%	≤0.001
Jameel et al 2012 [31]	43.77	31.26	56.28	99.1%	≤0.001
Ali et al 2007 [32]	44.16	31.85	56.47	99.1%	≤0.001
Khalil et al 2014 [33]	42.94	30.22	55.66	92.2%	≤0.001
Mallhi et al 2017 [34]	44.83	31.17	56.48	99.1%	≤0.001
Budastra et al 2009 [36]	44.19	31.91	56.47	99.1%	≤0.001
Liu et al 2013 [37]	40.67	28.87	52.47	99.1%	≤0.001
Bashir et al 2015 [39]	44.37	32.26	56.47	99.1%	≤0.001
Shah et al 2005 [41]	42.31	30.03	54.59	99.2%	≤0.001
Khan et al 2020 [52]	43.75	31.28	56.23	99.2%	≤0.001
Combined	42.91	30.95	54.87	99.1%	≤0.001

**Table 3:** Sensitivity analysis of the included studies to estimate the magnitude of prolonged PT among patients with dengue virus infection.

Study omitted	Point Estimate	95% Conf. Interval		Heterogeneity	
		Lower	Upper	I^2^	P-Value
Pranesh 2020 [13]	14.45	9.10	19.81	96.8%	≤0.001
Barbosa et al 2019 [17]	15.23	9.75	20.71	96.9%	≤0.001
Hassan et al 2018 [18]	16.71	10.86	22.56	97.5%	≤0.001
Dhooria et al 2008 [27]	17.44	11.60	23.38	97.1%	≤0.001
Jameel et al 2012 [31]	15.92	10.28	21.55	96.9%	≤0.001
Ali et al 2007 [32]	17.56	11.66	23.47	96.7%	≤0.001
Khalil et al 2014 [33]	16.88	10.78	222.98	97.2%	≤0.001
Mallhi et al 2017 [34]	15.00	10.22	19.78	95.6%	≤0.001
Budastra et al 2009 [36]	16.58	10.79	22.37	97.2%	≤0.001
Liu et al 2013 [37]	17.62	11.81	23.44	96.8%	≤0.001
Bashir et al 2015 [39]	17.09	11.05	23.13	97.2%	≤0.001
Shah et al 2005 [41]	16.02	10.33	21.70	97.2%	≤0.001
Khan et al 2020 [52]	17.30	11.23	23.37	97.1%	≤0.001
Combined	16.48	10.95	21.01	97.0%	≤0.001

### Meta-regression

We have done meta-regression by considering the continuous covariate year of publication. The result of the meta-regression showed that the overall magnitude of prolonged PT and APTT among dengue fever patients was not associated with year of publication (Table 4).

**Table 4:** Meta-Regression.

Variables	APTT		PT	
	Coefficient	P-value	Coefficient	P-value
Publication year	0.05	0.434	0.108	0.145

## Discussion

A total of 42 studies were included in this systematic review and meta-analysis. Accordingly, the magnitude of prolonged APTT and PT was 42.91% (95% CI: 30.95, 54.87) I^2^ = 99.1% and 16.48% (95% CI: 10.95, 22.01) I^2^ = 97.0%, respectively. We have used 9 studies that reported prolonged APTT in the form of mean± SD. Accordingly, the mean APTT in the included studies was 41.65 ±7.39 (95% CI: 41.29, 42.01). Besides, using 6 studies, the mean PT value was 15.17±2.47 (95% CI: 15.04, 15.30).

Dengue infection is characterized by increased vascular permeability and abnormal hemostasis [12]. Platelet function is also abnormal in dengue infections [61]. Coagulopathy is multifactorial and may be due to low platelets, deranged PT, APTT, and hepatitis [33]. Damage to liver cells decreases the coagulation factor synthesis and this, in turn, can alter the PT and APTT systems [24]. The APTT and PT are indicators of the intrinsic and extrinsic pathways of the coagulation system. Prolongation of PT and APTT might be caused either by the down-regulation of synthesis of specific factors or by an increase in consumption of specific factors [19]. The non-structural protein 1 (NS1) of the dengue virus can bind both to thrombin and prothrombin. Binding to thrombin will not make any changes whereas prothrombin activation is inhibited. This can explain changes in APTT occur early before antibodies are formed [62]. Coagulopathy as indicated by prolongation of APTT shows an abnormality in the intrinsic pathway of coagulation which lasts only for few days during the disease course [23]. APTT prolongation in the DF patients is caused by a lack of intrinsic pathway probably due to impaired synthesis of coagulation factor [10]. Reductions in the levels of specific coagulation factors such as II, V, VII, VIII, IX, X, antithrombin, and alpha-2 antiplasmin have been reported in DHF patients [18]. The IL-6 plays its role in down-regulating the synthesis of factor XII, the first factor to initiate the intrinsic pathway of coagulation [23].

The other laboratory abnormality determined in this study was the magnitude of thrombocytopenia among dengue fever patients. Twenty-six studies were included to determine the pooled prevalence of thrombocytopenia in dengue fever patients. Accordingly, 70.29% (95% CI: 62.69, 77.89) of those patients had thrombocytopenia. Platelet counts begin to fall during the febrile stage and reach their nadir during the toxic stage [63]. The development of thrombocytopenia in dengue fever infection might be due to depression of bone marrow observed in the acute stage of dengue virus infection. Other explanations are direct infection of the megakaryocytes by virus leading to increased destruction of the platelets or the presence of antibodies directed against the platelets [35]. The third mechanism is increased platelet consumption from the interaction between platelets and endothelial cells infected with dengue virus was demonstrated in vitro and suggested that some dengue-injured endothelial cells might promote platelet adherence and lysis [64].

The subgroup analysis in this review showed that children experienced prolonged APTT than other age groups. This might be explained that dengue infection was thought to be a disease that mostly affected children. DHF has been described as a disease that almost exclusively affects children age <16 years [65]. A study conducted by Hamond et al showed that infants and children 4–6 years of age were significantly more likely than adults to develop DHF/DSS or manifestations of severe clinical illness [66]. The presence of shock and hemorrhagic manifestations during infancy can be attributed to passively transferred circulating antibodies from the mother [67]. However, some studies have reported that the age distribution of this disease has shifted to older age groups [68]. The major burden of disease in infants and children 5 to 9 years of age can be expected in a country that has been endemic for dengue for a long period of time. However, countries with a shorter or non-endemic history of dengue report cases principally in the adolescent and adult population [66]. In this study, in the contrary to the result of prolonged APTT, the results of the prolonged PT was higher in adults than in the other age groups.

This study had some limitations to be considered. The study did not explore potential factors contributing to prolonged APTT, prolonged PT, and also thrombocytopenia in dengue fever patients. Besides this study didn’t summarize factor deficiencies in dengue fever patients. We also included articles published in the English language only.

## Conclusion

The result of this study showed that there is high magnitude of prolonged APTT and PT in dengue fever patients. Therefore, screening and early correction of coagulation abnormalities may be helpful to reduce further complications in those patients.

## Supporting information

S1 PRISMA Checklist (DOCX)

S1 PubMed search strategy (DOCX)

S1 Quality appraisal (DOCX)
