# Supplementary material for: Coagulation abnormalities in Dengue fever infection: A systematic review and meta-analysis
Source: PLoS Negl Trop Dis. 2021 Aug 18;15(8):e0009666. doi: 10.1371/journal.pntd.0009666 (PMC8372965; doi:10.1371/journal.pntd.0009666)
Supplement: S1 PubMed search strategy — (DOCX) [file pntd.0009666.s002.docx]

| Search | Search terms | Hits |
| --- | --- | --- |
| 1 | (((((((Coagulation abnormalities[Text Word]) OR (Coagulation profiles[Text Word])) OR (Prothrombin time[Text Word])) OR (Partial thromboplastin time[Text Word])) OR (Hemostatic derangement[Text Word])) ) OR (Hematological profiles[Text Word])) OR (Thrombocytopenia[Text Word]) | 89,082 |
| 2 | ((((((Coagulation abnormalities[MeSH Terms]) OR (Coagulation profiles[MeSH Terms])) OR (Prothrombin time[MeSH Terms])) OR (Partial thromboplastin time[MeSH Terms])) OR (Hemostatic derangement[MeSH Terms])) OR (Hematological profiles[MeSH Terms])) OR (Thrombocytopenia[MeSH Terms]) | 67,458 |
| 3 | #1 OR #2 | 108,584 |
| 4 | ((Dengue fever[Text Word]) OR (Dengue hemorrhagic fever[Text Word])) OR (Dengue shock syndrome[Text Word]) | 6,564 |
| 5 | #3 AND #4 | 584 |

S1 PubMed search strategy for the magnitude of coagulation abnormalities among patients with dengue fever-2021
