# Supplementary material for: Coagulation abnormalities in Dengue fever infection: A systematic review and meta-analysis
Source: PLoS Negl Trop Dis. 2021 Aug 18;15(8):e0009666. doi: 10.1371/journal.pntd.0009666 (PMC8372965; doi:10.1371/journal.pntd.0009666)
Supplement: S1 Quality appraisal — (DOCX) [file pntd.0009666.s003.docx]

S1 Quality assessment of the studies included in systematic review and meta-analysis of coagulation abnormalities among dengue fever patients

| Author, year of publication | Q1 | Q2 | Q3 | Q4 | Q5 | Q6 | Q7 | Q8 | Q9 | Q10 | Q11 | Total score |
| --- | --- | --- | --- | --- | --- | --- | --- | --- | --- | --- | --- | --- |
| Hassan et al 2018 [1] | Y | Y | Y | NA | Y | Y | Y | Y | Y |  | | 8 |
| Vijayaraghavan et al 2020 [2] | Y | Y | Y | NA | Y | Y | Y | Y | Y |  | | 8 |
| Kannan et al 2014 [3] | Y | Y | NA | Y | Y | Y | Y | NA | Y |  | | 7 |
| Balakrishnan et al 2017 [4] | Y | Y | Y | NA | Y | Y | Y | NA | Y |  | | 7 |
| Yashaswini et al 2017 [5] | Y | Y | NA | NA | Y | Y | Y | Y | Y |  | | 7 |
| Kadadavar et al 2019 [6] | Y | Y | NA | NA | Y | Y | Y | Y | Y |  | | 7 |
| Dhooria et al 2008 [7] | Y | Y | NA | Y | Y | Y | Y | Y | Y |  | | 8 |
| Kavitha et al 2020 [8] | Y | Y | Y | NA | Y | Y | Y | Y | Y |  | | 8 |
| Kalori et al 2011 [9] | Y | Y | Y | NA | Y | Y | Y | NA | Y |  | | 7 |
| Hamsa et al 2019 [10] | Y | Y | Y | NA | Y | NA | Y | Y | Y |  | | 7 |
| Jameel et al 2012 [11] | Y | Y | Y | Y | NA | Y | Y | Y | Y |  | | 8 |
| Ali et al 2007 [12] | Y | Y | Y | NA | Y | Y | Y | NA | Y |  | | 7 |
| Khalil et al 2014 [13] | Y | Y | Y | Y | Y | Y | Y | Y | Y |  | | 9 |
| Mallhi et al 2017 [14] | Y | Y | Y | Y | Y | Y | Y | Y | Y |  | | 9 |
| Ayyub et al 2006 [15] | Y | Y | NA | Y | Y | Y | Y | Y | Y |  | | 8 |
| Budastra et al 2009 [16] | Y | Y | Y | Y | Y | Y | Y | Y | Y |  | | 9 |
| Liu et al 2013 [17] | Y | Y | Y | Y | Y | Y | Y | Y | Y |  | | 9 |
| Kulasinghe et al 2016 [18] | Y | Y | Y | NA | Y | Y | Y | Y | Y |  | | 8 |
| Bashir et al 2015 [19] | Y | Y | Y | NA | Y | Y | Y | Y | Y |  | | 8 |
| Khan et al 2020 [20] | Y | Y | Y | Y | NA | Y | Y | NA | Y |  | | 7 |
| Shah et al 2005 [21] | Y | Y | Y | Y | Y | Y | Y | Y | NA |  | | 8 |
| Ghalige et al 2014 [22] | Y | Y | Y | Y | Y | Y | Y | NA | Y |  | | 8 |
| Selvan et al 2015 [23] | Y | Y | Y | NA | Y | Y | Y | Y | NA |  | | 7 |
| Khan et al 2014 [24] | Y | Y | Y | NA | Y | Y | Y | NA | Y |  | | 7 |
| Tulara et al 2019 [25] | Y | Y | Y | NA | Y | Y | Y | NA | Y |  | | 7 |
| Kumari et al 2020 [26] | Y | Y | Y | Y | Y | NA | Y | Y | Y |  | | 8 |
| Rai et al 2019 [27] | Y | Y | Y | NA | Y | Y | Y | NA | Y |  | | 7 |
| Tewari et al 2018 [28] | Y | Y | Y | NA | Y | Y | Y | Y | Y |  | | 8 |
| Chairulfatah et al 2003 [29] | Y | Y | Y | NA | Y | Y | Y | NA | NA |  | | 6 |
| Khan et al 2014 [30] | Y | Y | Y | Y | Y | NA | Y | NA | NA |  | | 6 |
| Patel et al 2020 [31] | Y | Y | Y | Y | NA | NA | Y | NA | Y |  | | 6 |
| Almas et al 2010 [32] | Y | Y | Y | Y | Y | Y | Y | NA | Y |  | | 8 |
| Prabhavathi et al 2017 [33] | Y | Y | Y | NA | Y | NA | Y | NA | Y |  | | 6 |
| Hsieh et al 2016 [34] | Y | Y | Y | Y | Y | Y | Y | Y | Y |  | | 9 |
| Kumar et al 2017 [35] | Y | Y | Y | NA | Y | Y | Y | Y | Y |  | | 8 |
| Ho et al 2013 [36] | Y | Y | Y | Y | Y | Y | Y | Y | Y |  | | 9 |
| Bandaru et al 2019 [37] | Y | Y | Y | Y | NA | Y | Y | NA | Y |  | | 7 |
| Vinoj M 2019 [38] | Y | Y | Y | Y | Y | Y | Y | Y | Y |  | | 9 |
| Case-control studies | | | | | | | | | | |  |  |
| Pranesh 2020 [39] | Y | Y | Y | Y | Y | Y | NA | Y | Y | Y |  | 9 |
| Cohort studies | | | | | | | | | | | | |
| Barbosa et al 2019 [40] | Y | Y | Y | NA | Y | Y | Y | Y | Y | Y | Y | 10 |
| Tong et al 2007 [41] | Y | Y | Y | Y | Y | Y | Y | Y | Y | NA | NA | 9 |
| Castilho et al 2020 [42] | Y | Y | Y | Y | Y | Y | Y | Y | Y | NA | Y | 10 |

**Key:** **Y**= Yes; **NR**= Not reported, **NA**=Not appropriate

**Question codes (prevalence study):**

1. Was the sample frame appropriate to address the target population?

2. Were study participants sampled in an appropriate way?

3. Was the sample size adequate?

4. Were the study subjects and the setting described in detail?

5. Was the data analysis conducted with sufficient coverage of the identified sample?

6. Were valid methods used for the identification of the condition?

7. Was the condition measured in a standard, reliable way for all participants?

8. Was there appropriate statistical analysis?

9. was the response rate adequate, and if not, was the low response rate managed appropriately?

**Question codes (case-control studies)**

1. Were the groups comparable other than the presence of disease in cases or the absence of disease in controls?

2. Were cases and controls matched appropriately?

3. Were the same criteria used for identification of cases and controls?

4. Was exposure measured in a standard, valid and reliable way?

5. Was exposure measured in the same way for cases and controls?

6. Were confounding factors identified?

7. Were strategies to deal with confounding factors stated?

8. Were outcomes assessed in a standard, valid and reliable way for cases and controls?

9. Was the exposure period of interest long enough to be meaningful?

10. Was appropriate statistical analysis used?

**Question codes for cohort studies**

1. Were the two groups similar and recruited from the same population?

2. Were the exposures measured similarly to assign people to both exposed and unexposed groups?

3. Was the exposure measured in a valid and reliable way?

4. Were confounding factors identified?

5. Were strategies to deal with confounding factors stated?

6. Were the groups/participants free of the outcome at the start of the study (or at the moment of exposure)?

7. Were the outcomes measured in a valid and reliable way?

8. Was the follow up time reported and sufficient to be long enough for outcomes to occur?

9. Was follow up complete, and if not, were the reasons to loss to follow up described and explored?

10. Were strategies to address incomplete follow up utilized?

11. Was appropriate statistical analysis used?

**References**

1. Hassan J, Borhany M, Abid M, Zaidi U, Fatima N, Shamsi T. Coagulation abnormalities in dengue and dengue haemorrhagic fever patients. Transfusion Medicine. 2020;30(1):46-50.

2. Vijayaraghavan YT, Weu F, Palile H. Predictors of Dengue Shock Syndrome: APTT Elevation as a Risk Factor in Children with Dengue Fever. J Infect Dis Epidemiol. 2020;6:111.

3. Kannan A, Narayanan KS, Sasikumar S, Philipose J, Surendran SA. Coagulopathy in dengue fever patients. Int J Res Med Sci. 2014;2(3):1070-2.

4. Vijayakumar Balakrishnan* SL, Lalitha Kailas. The coagulation profile of children admitted with dengue fever and

correlation with clinical severity. International Journal of Contemporary Pediatrics. 2017;4(6):5. doi: http://dx.doi.org/10.18203/2349-3291.ijcp20174741.

5. Yashaswini L. Priya. A study of hematological parameters and requirement of platelet transfusion in dengue fever. Int J Adv Med. 2017;4(6):1668.

6. Kadadavar SS, Lokapur V, Nadig D, Prabhu M, Masur D. Hematological parameters in dengue fever: A study in tertiary care hospital. Indian Journal of Pathology and Oncology. 2020;7(2):218-22.

7. Dhooria GS, Bhat D, Bains HS. Clinical profile and outcome in children of dengue hemorrhagic fever in North India. 2008.

8. Kavitha R, Clarin JD. A Study of Incidence and Significance of Section Coagulopathy among Dengue Patients Admitted in a Tertiary Care Hospital at Tirunelveli, Tamil Nadu, India. 2020.

9. Karoli R, Fatima J, Siddiqi Z, Kazmi KI, Sultania AR. Clinical profile of dengue infection at a teaching hospital in North India. The Journal of Infection in Developing Countries. 2012;6(07):551-4.

10. Hamsa B.T. SSV, Prabhakar K., Raveesha A., Manoj A.G. Significance of APTT as early predictor of bleeding in comparisonto thrombocytopenia in dengue virus infection. International Journal of Research in Medical Sciences| January2019| Vol 7| Issue 1Page 67International Journal of Research in Medical Sciences. 2019;7(1):4. doi: http://dx.doi.org/10.18203/2320-6012.ijrms20185094.

11. Jameel T, Mehmood K, Mujtaba G, Choudhry N, Afzal N, Paul RF. Changing haematological parameters in dengue viral infections. Journal of Ayub Medical College Abbottabad. 2012;24(1):3-6.

12. Ali N, Usman M, Syed N, Khurshid M. Haemorrhagic manifestations and utility of haematological parameters in dengue fever: a tertiary care centre experience at Karachi. Scandinavian journal of infectious diseases. 2007;39(11-12):1025-8.

13. Khalil MAM, Tan J, Khalil MAU, Awan S, Rangasami M. Predictors of hospital stay and mortality in dengue virus infection-experience from Aga Khan University Hospital Pakistan. BMC research notes. 2014;7(1):1-7.

14. Mallhi TH, Khan AH, Sarriff A, Adnan AS, Khan YH. Determinants of mortality and prolonged hospital stay among dengue patients attending tertiary care hospital: a cross-sectional retrospective analysis. BMJ open. 2017;7(7):e016805.

15. Ayyub M, Khazindar AM, Lubbad EH, Barlas S, Alfi AY, Al-Ukayli S. Characteristics of dengue fever in a large public hospital, Jeddah, Saudi Arabia. Journal of Ayub Medical College Abbottabad. 2006;18(2):9-13.

16. Budastra I, Arhana B, Mudita I. Plasma prothrombin time and activated partial thromboplastin time as predictors of bleeding manifestations during dengue hemorrhagic fever. Paediatrica Indonesiana. 2009;49(2):69-74.

17. Liu J-W, Lee I-K, Wang L, Chen R-F, Yang KD. The usefulness of clinical-practice-based laboratory data in facilitating the diagnosis of dengue illness. BioMed research international. 2013;2013.

18. Kulasinghe S, Ediriweera R, Kumara P. Association of abnormal coagulation tests with dengue virus infection and their significance as early predictors of fluid leakage and bleeding. Sri Lanka Journal of Child Health. 2016;45(3).

19. Mohammed BAB. Deranged liver among Sudanese patients with dengue virus infection in Port Sudan Teaching Hospital. Sudan Journal of Medical Sciences. 2017;12(3):187-97.

20. Khan S, Baki MA, Ahmed T, Mollah MAH. Clinical and laboratory profile of dengue fever in hospitalized children in a tertiary care hospital in Bangladesh. BIRDEM Medical Journal. 2020;10(3):200-3.

21. Shah I, Katira B. Clinical and Laboratory Abnormalities due to Dengue in Hospitalized Children in Mumbai in 2004. 2005.

22. Ghalige SS, Reddy CU, Prakash S, Aradhya GH. Bleeding risk in Dengue fever: A clinico-laboratory profile study. RGUHS Journal of Medical Sciences. 2014;4(4):189-92.

23. Selvan T, Souza JLD, Giridhar NS, Kumar M. Prevalence and severity of Thrombocytopenia in Dengue fever in children. Scholars journal of Applied Medical Sciences (SJAMS). 2015;3(5D):2068-70.

24. Khan MU, Rehman R, Gulfraz M, Latif W. Incidence of thrombocytopenia in seropositive dengue patients. International Journal of Medicine and Medical Sciences. 2014;6(4):113-6.

25. Tulara NK. Dengue fever and thrombocytopenia–A prospective Observational Study at Tertiary Care Centre. Eastern Journal of Medical Sciences. 2019:45-8.

26. Kumari S, Makwana M, Mourya HK, Mitharwal R, Ram S, Meena A, et al. An observational study to determine the incidence and the clinico-epidemiologic profile of dengue fever in paediatric age group presenting to a tertiary care centre in Western Rajasthan, India. 2020.

27. Rai A, Azad S, Nautiyal S, Acharya S. Correlation between hematological and serological parameters in dengue patients-an analysis of 2022 cases.

28. Tewari K, Tewari VV, Mehta R. Clinical and hematological profile of patients with dengue fever at a tertiary care hospital–an observational study. Mediterranean journal of hematology and infectious diseases. 2018;10(1).

29. Chairulfatah A, Setiabudi D, Agoes R, Colebunders R. Thrombocytopenia and Platelet Trasnfusions in Dengue Haemorrhagic Fever and Dengue Shock Syndrome. 2003.

30. Khan DM, Kuppusamy K, Sumathi S, Mrinalini V. Evaluation of thrombocytopenia in dengue infection along with seasonal variation in rural Melmaruvathur. Journal of clinical and diagnostic research: JCDR. 2014;8(1):39.

31. Patel MK, Patel HJ. Assessment of clinical and hematological profile in dengue fever. International Journal of Advances in Medicine. 2020;7(9):1418.

32. Almas A, Parkash O, Akhter J. Clinical factors associated with mortality in dengue infection at a tertiary care center. Southeast Asian J Trop Med Public Health. 2010;41(2):333-40.

33. Prabhavathi R, Madhusudan S, Suman M, Govindaraj M, Puttaswamy M. Study of clinical and laboratory predictive markers of dengue fever and severe dengue in children. J Pediatr Res. 2017;4(6):397-404.

34. Hsieh C-C, Cia C-T, Lee J-C, Sung J-M, Lee N-Y, Chen P-L, et al. A cohort study of adult patients with severe dengue in Taiwanese intensive care units: the elderly and APTT prolongation matter for prognosis. PLoS neglected tropical diseases. 2017;11(1):e0005270.

35. Kumar BV, Simna L, Kalpana D, Kailas L. Clinical profile and outcome of children admitted with dengue fever in a tertiary care hospital in South India. Indian Journal of Child Health. 2018;5(1):32-7.

36. Ho T-S, Wang S-M, Lin Y-S, Liu C-C. Clinical and laboratory predictive markers for acute dengue infection. Journal of biomedical science. 2013;20(1):1-8.

37. Bandaru AK, Vanumu DS. Correlation of liver indices with thrombocytopenia in dengue infected children.

38. Vinoj M. Association of Abnormal Coagulation Profile and Liver Enzymes with Dengue Infection and Their Significance as Predictors of Assessing Severity of Disease: Madurai Medical College, Madurai; 2019.

39. Pranesh S. A Study of Activated Partial Thromboplastin Time and Prothrombin Time as Predictors for Impaired Coagulation

40. Barbosa ACN, Montalvão SAL, Barbosa KGN, Colella MP, Annichino-Bizzacchi JM, Ozelo MC, et al. Prolonged APTT of unknown etiology: A systematic evaluation of causes and laboratory resource use in an outpatient hemostasis academic unit. Research and practice in thrombosis and haemostasis. 2019;3(4):749-57.

41. Tong S, Aziz N, Chin G. Predictive value of thrombocytopaenia in the diagnosis of dengue infection in outpatient settings. The Medical journal of Malaysia. 2007;62(5):390-3.

42. Castilho BM, Silva MT, Freitas AR, Fulone I, Lopes LC. Factors associated with thrombocytopenia in patients with dengue fever: a retrospective cohort study. BMJ open. 2020;10(9):e035120
